# Liquid chromatography-tandem mass spectrometry analysis for identification and quantification of antimicrobial compounds in distillery wastewater

**DOI:** 10.1016/j.mex.2021.101470

**Published:** 2021-07-24

**Authors:** Waner Hou, Jiayin Ling, Yanbin Xu, Kailing Li, Fei Wang

**Affiliations:** a Guangdong Provincial Key Laboratory of Environmental Health and Land Resource, Zhaoqing University, Zhaoqing 526061, Guangdong, China; b Analysis and Test Center, Guangdong University of Technology, Guangzhou 510006, China; c School of Environmental Science and Engineering, Guangdong University of Technology, Guangzhou 510006, China

**Keywords:** Liquid chromatography-tandem mass spectrometry, Qualitative analysis, Quantitative analysis, Distillery wastewater, Lactic acid, Succinic acid, Cinnamic acid, Phenyllactic acid, Methylmalonic acid, Acetophenone

## Abstract

A high-resolution mass spectrometry (HR-MS) method was developed to analyze and identify small molecule compounds in distillery wastewater. According to identification confidence levels, 4 levels of compounds were identified. The five antimicrobial compounds (lactic acid, succinic acid, acetophenone, cinnamic acid, and phenyllactic acid), which shown in high concentrations, were at the highest level of confidence (level 1, confirmed structure). Thus, a rapid and sensitive liquid chromatography-tandem mass spectrometry (LC-MS/MS) method was developed to simultaneously quantify these antimicrobial compounds. The analysis was performed in the selective reaction monitoring (SRM) mode via the electrospray ionization (ESI) source operating in the negative ionization mode. Linear calibration curves were obtained over the concentration range of 50–1000.0 ng/mL for succinic acid, acetophenone, cinnamic acid, phenyllactic acid, and 375–7500 ng/mL for lactic acid. Precision and recovery of the analytes were all satisfactory (relative standard deviation < 10%). The validated method was successfully applied to quantitative analysis of the five antimicrobial compounds in distillery wastewater.

• Analyze and identify 4 levels of small molecule compounds in distillery wastewater.
• Simple method for quantification of five antimicrobial compounds.
• Column temperature affected the lactic and succinic acid chromatographs significantly.

## Highlights

• Analyze and identify 4 levels of small molecule compounds in distillery wastewater.
• Simple method for quantification of five antimicrobial compounds.
• Column temperature affected the lactic and succinic acid chromatographs significantly.

## Graphical Abstract

**Figure:**
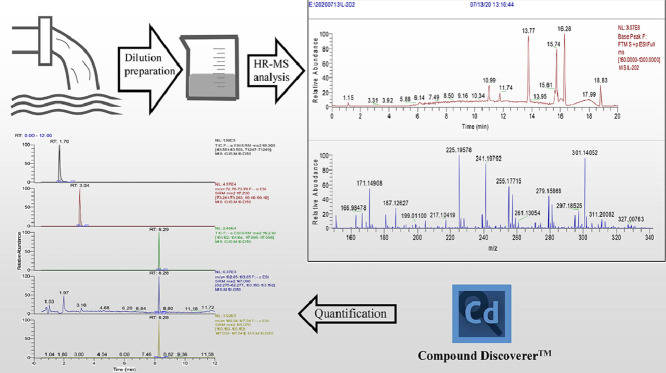

Specifications Table

**Table:** 

Subject Area:	*Environmental Science*
More specific subject area:	A *dvanced mass spectrometric analysis for environmental and food safety, Analytical chemistry, Wastewater analysis*
Method name:	*Liquid chromatography-tandem mass spectrometry analysis for identification and quantification of antimicrobial compounds in distillery wastewater*
Name and reference of original method:	*E. L. Schymanski, J. Jeon, R. Gulde, K. Fenner, M. Ruff, H. P. Singer, J. Hollender, Identifying Small Molecules via High Resolution Mass Spectrometry: Communicating Confidence. Environ. Sci. Technol., 48 (2014) 2097-2098*
Resource availability:	*CompoundDiscoverer 2.1 (Thermo Scientific), mzCloud database (Thermo Scientific,*http://www.mzcloud.org)

## *Method details

 

## Introduction

Distillery wastewater could cause many environment issues due to its high generation amount and high concentration of organics and nutrients [1]. Therefore, it is important to develop methods to analyze the composition of distillery wastewater to support the improvement of resource recovery and treatment process of distillery wastewater. In this study, a high-resolution mass spectrometry (HR-MS) method was developed to analyze and identify small molecules compounds in distillery wastewater and 4 levels of compounds were identified. And an effective and rapid method has been developed for simultaneous determination of lactic acid, succinic acid, acetophenone, cinnamic acid and phenyllactic acid (the five identified major antimicrobial compounds) in the distillery wastewater using a simple one-step sample dilution preparation couple with UPLC-MS/MS.

## Materials and reagents

Lactic acid, succinic acid, acetophenone, cinnamic acid and phenyllactic acid were purchased from the Sigma-Aldrich Company Ltd.

HPLC-grade formic acid and MS-grade methanol purchased from Merck (Darmstadt, Germany) were used for HPLC analysis and sample preparation.

## Preparation of standard solution and distillery wastewater samples

Concentrated stock solutions of analytes were prepared by dissolving the appropriate amount of the standard samples in 50% methanol at a concentration of 1 mg/mL. And then it was further diluted with acetonitrile to form a series of working solutions used to prepare the calibration curve. All the solutions were stored at –20 °C.

A10 μl of the distillery wastewater sample was added with a 20 mL of 50% methanol solution was added. Then, the mixture was vortexed for 2 min and centrifugation at 13,000 rpm for 10 min at 4 °C. Subsequently, the supernatant liquor was transferred to centrifugation at 13,000 rpm for 5 min at 4 °C again, then the supernatant liquor was injected into the HPLC-MS/MS for analysis.

## Identification of antimicrobial compounds by HR-MS

### Analytical instrumentation

The LC–MS/MS system used was a Thermo Scientific Ultimate 3000 liquid phase system equipped with Q Exactive Orbitrap and an electrospray ionization source. A volume of 2 μl sample was injected to a Hypersil Gold C18 column (100 × 2.1 mm, 1.9 μm, Thermo Scientific) at 20 °C. The LC flow was set to 250 μl/min using H_2_O (0.1% formic acid) and methanol as eluents. The gradient elution started with 98% H_2_O for 2 min and was changed to 95% methanol over the course of 13 min, maintained for 3 min, then returned to 98% H_2_O within 0.1 min, and equilibrated for 1.9 min prior to the next injection. The heated electrospray ionization source had a capillary temperature of 350 °C.

Both positive and negative electrospray ionization were employed to obtain MS signals of analytes with spray voltages of +3.5 kV and -2.5 kV, respectively. Sheath gas flow rate, aux gas flow rate and sweep gas flow rate were set to 40, 10 and 0 (arbitrary units), respectively. Capillary temperature and aux gas heater temperature were set to 320 °C and 350 °C, respectively. The MS was set at full scan mode and acquire targeted first MS signals in at 70,000 fwhm and targeted MS/MS scan was set at a resolution of 175,00 fwhm with isolation width of 2.0 m/z. The instrument would automatically switch the positive and negative ion scanning mode and the scan mode was chosen as full MS scan-dd MS2 and acquire first MS signals at 70,000 fwhm and targeted MS/MS scan was set at a resolution of 175,00 fwhm with isolation width of 2.0 m/z. Meanwhile, the m/z scan range was 70–700.

### Data processing

**Fig. 1:**
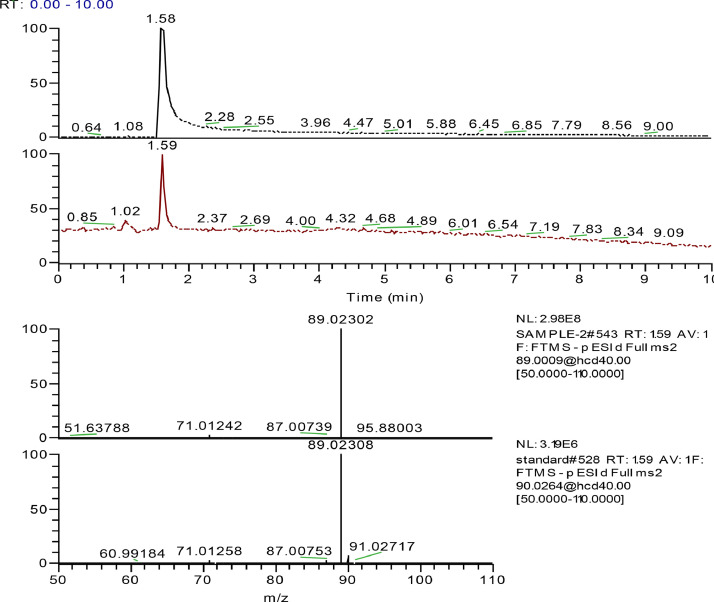
The extract chromatogram and MS/MS of lactic acid in the sample (top) compared with reference standards (bottom).

Peak detection and alignment of the LC−MS data were performed using Compound Discoverer 2.0 (Thermo Scientific) to obtain a peak list with peak areas, molecular weight, and retention time with the following settings: S/N threshold, 3; mass tolerance, 10 ppm; minimum peak intensity, 1 × 10^5^. With the application of the software, a possible molecular formula fitting the exact mass and isotope patterns was calculated. Furthermore, the MS/MS fragments were compared to the mzCloud database. Fig. 1 and S1-S4 (in the supplementary materials) show how compounds were identified. As can be seen, the MS and, MS/MS information and retention time of the unknown compound were highly consistent with the reference substance.

According to Identification confidence levels reported by Schymanski et al. [2], 4 levels of unknown compound were classified inTable 2.

**Table 1:** Gradient elution time program for mobile phase for qualitative analysis in LC-MS/MS.

Time (min)	%A(0.1% formic acid)	%B (methanol)
0	98	2
2	98	2
16	5	95
18	5	95
18.1	98	2
20	98	2

**Table 2:** Identification confidence levels according to Schymanski et al. [13].

Level	Identification confidence	Minimum data requirements
1	Confirmed structure by reference standard	MS, MS2, RT, reference Std.
2	Probable structure by library spectrum match	MS, MS2, library MS2
3	Tentative candidates(s)	MS, MS2, Exp. data
4	Unequivocal molecular formula	MS isotope/adduct

Table 3 showed the compounds contained in rice spirit distillery wastewater identified with four different confidence levels by HR-MS. Lactic acid, succinic acid, L-phenylalanine, caffeine, adenosine, D(+)-phenyllactic acid, DL-arginine, acetophenone and cinnamic acid were confirmed using the standard compounds. The MS, MS/MS and retention time compared with reference standards (lactic acid, succinic acid, acetophenone, cinnamic acid and phenyllactic acid) were shown in Fig. 1 and S1-4. Approximate 60 compounds were converged to level 2 in the identified top 100 most abundant compounds (based on peak area). Their MS/MS fragments were compared to the mzCloud database and had a direct matching. In Fig. S5, Υ-aminobutyric acid, L-glutamic acid, proline and D-(+)-pyroglutamic acid were chosen as representatives to show the MS2 spectrum comparison between the sample and mzCloud library. Fig. S6 was the chromatogram and ms2 spectrum of extract mass 132.1019, indicated the existence of leucine or isoleucine. In level 4, a possible molecular formula fitting the exact mass and isotope patterns was calculated.

**Table 3:** Compounds contained in the rice spirit distillery wastewater identified with four different confidence levels by HR-MS (the top 100 most abundant compounds based on peak area).

No.	Name	Formula	Molecular Weight	RT [min]	Area	Identification confidence levels
1	Lactic acid	C3 H6 O3	90.03169	1.58	3E+10	1
2	Phenyllactic acid	C9H10O3	166.063	8.53	4E+09	1
3	Succinic acid	C4 H6 O4	118.0266	2.85	1E+09	1
4	Citraconic acid	C5 H6 O4	84.01995	1.85	1E+09	2
5	L-Norleucine	C6 H13 N O2	131.0946	2.98	3E+08	3
6	Cinnamic acid	C9H8O2	148.0524	8.53	2E+08	1
7	Gluconic acid	C6 H12 O7	150.052	1.03	2E+08	2
8	L-Phenylalanine	C9 H11 N O2	165.0789	5.39	2E+08	1
9	Acetophenone	C8 H8 O	120.0575	4.26	9E+07	1
10	6-Hydroxycaproic acid	C6 H12 O3	86.07209	8.16	7E+07	2
11	D(+)-Phenyllactic acid	C9 H10 O3	120.0568	8.56	6E+07	2
12	Υ-Aminobutyric acid (GABA)	C4 H9 N O2	103.0635	1.11	6E+07	2
13	L-Leucine	C6 H13 N O2	131.0946	2.78	6E+07	3
14	2-Hydroxycinnamic acid	C9 H8 O3	164.0473	4.24	5E+07	4
15	Adenine	C5 H5 N5	135.0544	2.26	5E+07	2
16	DL-4-Hydroxyphenyllactic acid	C9 H10 O4	182.0574	7.07	4E+07	4
17	trans-3-Indoleacrylic acid	C11 H9 N O2	187.0631	7.22	4E+07	2
18	D-(+)-Proline	C5 H9 N O2	115.0634	1.16	3E+07	1
19	D-(+)-Pyroglutamic Acid	C5 H7 N O3	129.0426	2.34	3E+07	2
20	Guanine	C5 H5 N5 O	151.0493	2.28	3E+07	2
21	Methylmalonic acid	C4 H6 O4	118.0255	2.89	3E+07	2
22	2-Isopropylmalic acid	C7 H12 O5	116.0467	7.42	2E+07	4
23	D-α-Hydroxyglutaric acid	C5 H8 O5	148.0363	1.99	2E+07	4
24	Cyclo(leucylprolyl)	C11 H18 N2 O2	210.1365	8.42	1E+07	2
25	Dimethyl succinate	C6 H10 O4	146.0579	7.42	1E+07	4
26	Piceatannol	C14 H12 O4	244.0706	10.94	1E+07	2
27	Glycyl-L-leucine	C8 H16 N2 O3	188.1159	6.11	1E+07	2
28	L-(+)-Arginine	C6 H14 N4 O2	174.1114	1.04	1E+07	4
29	Spermidine	C7 H19 N3	128.1311	0.92	1E+07	4
30	L-(+)-Citrulline	C6 H13 N3 O3	158.0688	1.10	1E+07	2
31	Cyclo(phenylalanyl-prolyl)	C14 H16 N2 O2	244.1209	8.90	1E+07	2
32	Prolylleucine	C11 H20 N2 O3	456.2942	6.59	9E+06	2
33	Cytosine	C4 H5 N3 O	111.0433	1.25	9E+06	1
34	DL-Lysine	C6 H14 N2 O2	146.1053	1.76	9E+06	2
35	DL-Arginine	C6 H14 N4 O2	174.1114	1.57	9E+06	2
36	(15Z)-9,12,13-Trihydroxy-15-octadecenoic acid	C18 H34 O5	330.241	11.56	8E+06	2
37	2-Hydroxyvaleric acid	C5 H10 O3	72.0564	6.08	8E+06	4
38	Imidazolelactic acid	C6 H8 N2 O3	156.0531	1.33	7E+06	2
39	Valylproline	C10 H18 N2 O3	214.1315	4.02	6E+06	4
40	Hypoxanthine	C5 H4 N4 O	136.0382	3.03	6E+06	2
41	Ethyl oleate	C20 H38 O2	310.2865	14.57	6E+06	2
42	Indole-3-lactic acid	C11 H11 N O3	205.0737	8.81	6E+06	2
43	Histamine	C5 H9 N3	111.0797	0.98	6E+06	2
44	DL-Homoserine	C4 H9 N O3	87.032	1.05	5E+06	4
45	3-Methylcrotonylglycine	C7 H11 N O3	157.0736	5.70	5E+06	4
46	L(-)-Pipecolinic acid	C6 H11 N O2	129.0788	1.59	4E+06	2
47	D-(-)-Mannitol	C6 H14 O6	182.0783	1.04	4E+06	2
48	Caffeine	C8 H10 N4 O2	194.0802	7.89	4E+06	1
49	trans-Cinnamic acid	C9 H8 O2	148.0515	8.55	4E+06	4
50	L-Histidine	C6 H9 N3 O2	155.0691	1.00	4E+06	2
51	Trigonelline	C7 H7 N O2	137.0475	1.20	4E+06	2
52	L(+)-Ornithine	C5 H12 N2 O2	132.0897	0.98	4E+06	4
53	Daidzein	C15 H10 O4	254.0577	10.32	4E+06	2
54	D(+)-Phenyllactic acid	C9 H10 O3	166.0622	8.71	3E+06	2
55	(2R)-2,3-Dihydroxypropanoic acid	C3 H6 O4	106.0254	1.15	3E+06	4
56	α,α-Trehalose	C12 H22 O11	342.1163	1.09	3E+06	4
57	3-(2-Hydroxyethyl)indole	C10 H11 N O	129.0578	9.22	3E+06	2
58	Acetylcholine	C7 H15 N O2	145.11	1.49	3E+06	2
59	DL-Malic acid	C4 H6 O5	134.0204	1.40	3E+06	2
60	N-Acetylalanine	C5 H9 N O3	131.0575	2.98	3E+06	4
61	2-Hydroxy-4-methylthiobutanoic acid	C5 H10 O3 S	150.0342	6.28	3E+06	2
62	Uracil	C4 H4 N2 O2	112.0273	1.90	3E+06	2
63	D-(-)-Quinic acid	C7 H12 O6	192.0627	1.16	3E+06	2
64	Carnosine	C9 H14 N4 O3	226.1063	2.75	3E+06	2
65	Crotetamide	C12 H22 N2 O2	226.1678	10.15	3E+06	4
66	Uric acid	C5 H4 N4 O3	168.0278	3.06	2E+06	2
67	Acetylarginine	C8 H16 N4 O3	216.122	2.16	2E+06	2
68	L-(+)-Arginine	C6 H14 N4 O2	174.1114	1.26	2E+06	4
69	L-Ergothioneine	C9 H15 N3 O2 S	229.088	1.37	2E+06	4
70	Spermine	C10 H26 N4	202.2156	0.90	2E+06	2
71	N3,N4-Dimethyl-L-arginine	C8 H18 N4 O2	202.1426	1.63	2E+06	4
72	Nicotinic acid	C6 H5 N O2	123.032	1.94	2E+06	2
73	3-Ureidopropionic acid	C4 H8 N2 O3	132.0525	1.01	2E+06	2
74	2-Aminooctanedioic acid	C8 H15 N O4	143.0941	5.38	2E+06	4
75	Prolylglycine	C7 H12 N2 O3	172.0845	1.47	2E+06	2
76	9-Oxo-10(E),12(E)-octadecadienoic acid	C18 H30 O3	312.2296	11.56	2E+06	2
77	β-D-Glucopyranuronic acid	C6 H10 O7	194.0419	1.06	2E+06	4
78	5-Hydroxymethyl-2-furaldehyde	C6 H6 O3	126.0317	5.49	2E+06	2
79	Genistein	C15 H10 O5	270.0527	10.94	1E+06	2
80	Gallic acid	C7 H6 O5	170.0208	5.07	1E+06	2
81	2-Hydroxyvaleric acid	C5 H10 O3	118.0619	6.24	1E+06	4
82	2-(Acetylamino)hexanoic acid	C8 H15 N O3	173.1047	8.31	1E+06	2
83	7-Methylguanine	C6 H7 N5 O	165.0649	3.73	1E+06	2
84	2-Aminoadipic acid	C6 H11 N O4	161.0683	4.50	1E+06	4
85	Syringic acid	C9 H10 O5	198.0523	8.34	1E+06	4
86	Prolinamide	C5 H10 N2 O	97.05283	1.13	9E+05	2
87	Thymine	C5 H6 N2 O2	126.043	4.40	8E+05	4
88	N-Acetyl-L-phenylalanine	C11 H13 N O3	207.0893	8.64	8E+05	4
89	Ethyl palmitoleate	C18 H34 O2	282.2554	13.82	8E+05	2
90	3-Isopropylmalic acid	C7 H12 O5	176.0676	1.39	7E+05	4
91	Pseudouridine	C9 H12 N2 O6	244.0693	1.98	7E+05	2
92	Hydrolyzed fumonisin B1	C22 H47 N O5	405.3446	17.48	6E+05	4
93	Corchorifatty acid F	C18 H32 O5	328.2254	11.50	6E+05	2
94	Methylsuccinic acid	C5 H8 O4	132.0412	5.66	5E+05	2
95	D-(+)-Maltose	C12 H22 O11	364.0973	1.09	5E+05	2
96	N-Acetyl-L-tyrosine	C11 H13 N O4	223.0843	7.16	4E+05	4
97	Suberic acid	C8 H14 O4	174.0885	8.88	4E+05	2
98	(2R)-2,3-Dihydroxypropanoic acid	C3 H6 O4	106.0255	19.99	4E+05	4
99	Citroflex 4	C18 H32 O7	360.2141	13.33	3E+05	2
100	Glutaric acid	C5 H8 O4	132.0413	5.02	3E+05	2

## Quantification of antimicrobial compounds by LC-MS-MS

Among the compounds detected by LC-MS-MS, five of them are reported with antimicrobial activity and had relatively high concentrations in distillery wastewater, which may affect the resource recovery process for distillery wastewater via microorganisms. They are lactic acid [3], succinic acid [4], cinnamic acid [5], phenyllactic acid [6], acetophenone [7]. Therefore, an effective and rapid quantification method has been developed for these compounds in this study.

### Analytical instrumentation

The LC–MS/MS system consisted of a Thermo Scientific Ultimate 3000 liquid phase system and TSQ Endura triple quadrupole mass spectrometer with an electrospray ionization source. Chromatographic separation was achieved at 20°C on a Hypersil Gold C18 column (100 × 2.1 mm, 1.9 μm, Thermo Scientific) by gradient solution with 0–2 min, 98% mobile phase A;2–4 min, 98%→80% mobile phase A; 4–7 min, 80%→10% mobile phase A; 7–9 min, 10% mobile phase A;9.1–12 min, 98% mobile phase A, flowing at 0.25 mL/min. Eluent A was water containing 0.1% formic acid, and B was methanol. The injection volume was 2 μL.

To achieve better retention and separation of both hydrophilic and polar compounds, two chromatographic columns with different stationary phases (i.e. a HILIC column and a C18 column) were examined with various mobile phases and additives (i.e. formic acid, acetic acid and ammonium acetate). Additionally, gradients, flow rate and column temperatures (20–40 °C) were also explored. It was found that the chromatographs of lactic acid and succinic acid were significantly affected by the column temperatures. Based on the chromatograph of lactic acid and succinic acid under 20 °C and 30 °C (Fig. S7), 20 °C was selected as the column temperature to obtain a good peak shape.

The addition of ammonium acetate into formic acid water or acetic acid water as mobile phase significantly decreased peak responses while did not improve peak shapes simultaneously. Compared with acetic acid in water, formic acid in water as the mobile phase could narrow peak widths. Therefore, 0.1% formic acid in water was selected as one of the mobile phases. Though the two columns had similar performance in resolution, retention time and peak shape, Hypersil Gold C18 as chromatographic separation column was chosen rather than Syncronis Hilic column (for polar components) because the former one was more commonly used.

**Fig. 2:**
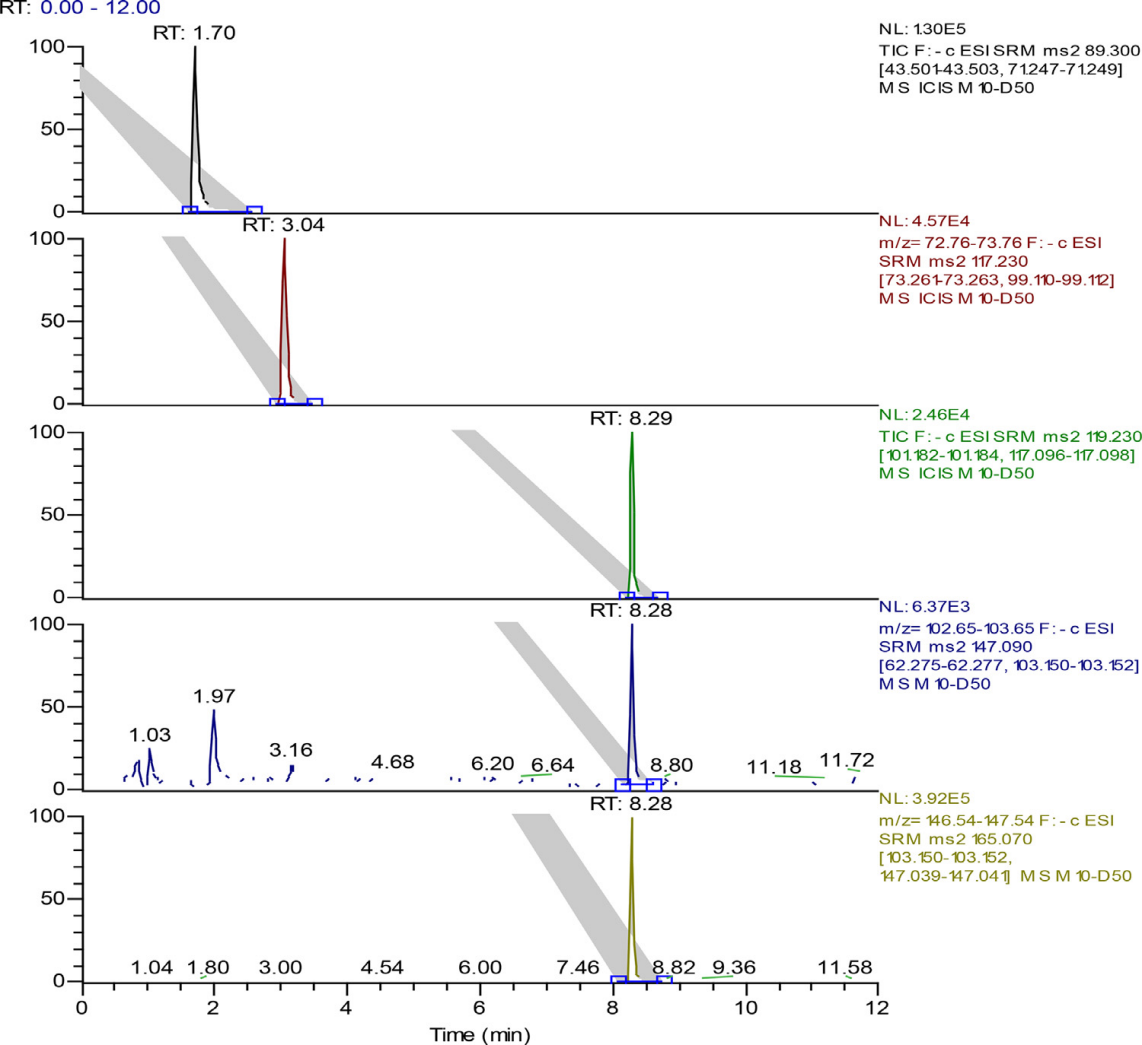
Typical chromatograms of the five analytes in distillery wastewater sample.

The mass spectrometer was operated in negative ion mode using SRM to detect the mass transitions. High purity nitrogen served as both nebulizing and drying gas. Compound-dependent parameters of the mass spectrometer were set as follows: spray voltage at 2500 V, capillary temperature at 320 °C, vaporizer temperature at 350 °C, sheath gas at 35 (Arb) and auxiliary gas at 10 (Arb). The parameters of SRM scan mode for each compound are shown in Table 4. Fig. 2 demonstrated typical chromatograms of the five analytes.

**Table 4:** MS/MS transitions and parameters for the analyses of the analytes.

Compounds	Polarity	Precursor (m/z)	Product (m/z)	Collision Energy (V)
Lactic acid	Negative	89.3	43.502(71.248*)	10.25
Succinic acid	Negative	117.23	73.262(99.111*)	10.25
Acetophenone	Negative	119.23	101.183(117.097*)	16.42
Cinnamic acid	Negative	147.09	62.276(103.151*)	10.25
Phenyllactic acid	Negative	165.07	103.151(147.04*)	10.25

Note: *qualitative ion.

### Validation of the method

The developed method was validated based on the recommendations published by FDA (Food and Drug Administration) [8]. The calibration curve consisted of five concentration levels. The linear regression of the areas of the analyte peaks versus the concentration were weighted with weighing factor 1/x^2^ (where x = concentration). The concentrations of the analyte were determined by interpolation from the calibration curve. Concentration of the standard sample in solvents with a signal-to-noise ratio (S/N) of 3 times is defined as instrumental detection limit. As shown in Table 5, all the analytes showed good linearity with regression coefficients (R^2^) values above 0.9981 (R > 0.9990). Linear ranges and IDL of the analytes were also shown in Table 5. The calibration curves of the five analytes were shown in Fig. S8.

**Table 5:** Linear range, R^2^ value and IDL of the analytes.

Compounds	Linear range (ng/mL)	R^2^	IDL (ng/mL)	Linear regression equation (Y, peak area; X, concentration)
Lactic acid	375–7500	0.9985	25	Y=61.991+3.7752*X
Succinic acid	50–1000	0.990	25	Y=-1108.29+65.2021*X
Acetophenone	50–1000	0.9983	0.5	Y=296.551+61.5398*X
Cinnamic acid	50–1000	0.9991	10	Y=73.5861+15.9079*X
Phenyllactic acid	50–1000	0.9984	1	Y=2286.28+1166.98*X

**Fig. 3:**
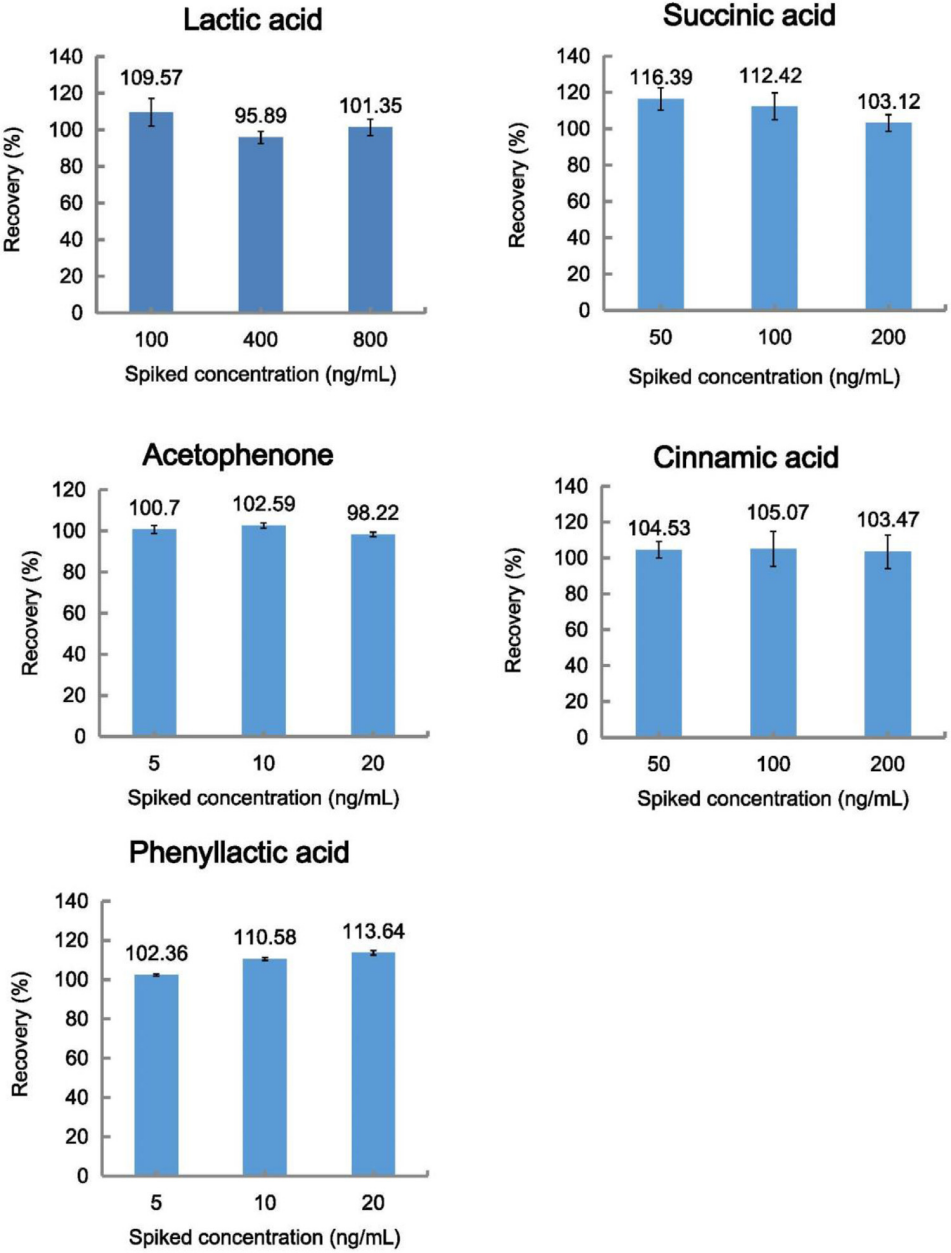
Recoveries of the five analytes at three concentration levels..

Three levels (low, medium and high) of organic acids were added to distillery wastewater samples to determine the precision (relative standard deviation, RSD) and extraction recovery (relative error, RE). Each level contained five validation samples. The recovery values of the five analytes at three concentration levels were shown in Fig. 3 and Table S1. All the recoveries were between 95.89% and 116.39% (RSD% < 9.80) at the three concentration levels of the analytes. These results were with the acceptance criteria and indicated that the method was accurate, reliable, and reproducible. Meanwhile, the wastewater samples were pretreated simply through dilution and centrifugation. These results of recoveries indicate that there was no significant matrix effect.

### Application

The established LC-MS/MS method was applied for determining the concentration of the five major antimicrobial compounds in distillery wastewater obtained from the rice spirit distillery located in Foshan city, Guangdong, Southern China. Table 6 was the quantitative analysis results of the five analytes in distillery wastewater.

**Table 6:** Quantitative analysis results of the five analytes in the distillery wastewater.

	Lactic acid	Succinic acid	Acetophenone	Cinnamic acid	Phenyllactic acid
Concentration (mg/L)	10,011–17,498	210–325	42–63	56–143	43–58

## Declaration of Competing Interest

The authors declare that they have no known competing financial interests or personal relationships that could have appeared to influence the work reported in this paper.

## Additional information

### Background information of this topic and method

Distillery wastewater could cause many environment issues such as eutrophication due to its high generation amount and high concentration of organics and nutrients [1]. The compounds contained in the distillery wastewater mainly come from the making process including pretreatment and hydrolysis of crops or fruits, fermentation, distillation and dehydration [9]. For effective treatment and resource recovery process of the distillery wastewater, it is necessary to identify the components in wastewater, especially the antimicrobial compounds that may affect the conventional biological treatment process and the resource recovery process for distillery wastewater via microorganisms such as microbial lipid (can be further converted to biodiesel) or biogas production from wastewater [10], [11], [12].

The increased availability and development of high resolution mass spectrometry (HR-MS) had dramatically improved the qualitative analysis of compounds in environmental (and other) samples. The elucidation of small molecules both parent compounds and their transformation products using HR-MS based non-target analysis is gaining in relevance in many fields (e.g. metabolomics, drug discovery, forensics) [13]. Therefore, a HR-MS analysis method for identification of small molecular compounds in distillery wastewater was developed in this study.

The quantitative analysis for high-concentration confirmed compounds (match the measured retention time and tandem mass spectrum with reference standards) are usually necessary for research purpose. In all the confirmed compounds, lactic acid, succinic acid, acetophenone, cinnamic acid and phenyllactic acid were closely related to our microbial contamination control mechanism research. At present, the main analysis methods of these organic acids are enzymatic method [14], gas chromatography (GC) [15,16], high performance liquid chromatography (HPLC) [17], [18], [19], ion-exclusion chromatography [20], liquid chromatography-tandem mass spectrometry analysis (LC-MS) [21], [22], [23] and so on. Enzymatic methods had a high limit of detection and GC required pre-treatment of derivatization. Though most organic acids could be detected by HPLC, the UV sensitivity is relatively low. LC-MS is widely used because of its high selectivity and sensitivity. Therefore, a LC-MS-MS method was developed for the quantification of the five antimicrobial compounds.

## Appendix B Supplementary materials

Image, application 1
