# Supplementary material for: Liquid chromatography-tandem mass spectrometry analysis for identification and quantification of antimicrobial compounds in distillery wastewater
Source: MethodsX. 2021 Jul 24;8:101470. doi: 10.1016/j.mex.2021.101470 (PMC8374650; doi:10.1016/j.mex.2021.101470)
Supplement: Supplementary file 1 [file mmc1.docx]

**Supplementary material and/or Additional information**

**Liquid chromatography-tandem mass spectrometry analysis for identification and quantification of antimicrobial compounds in distillery wastewater**

Waner Hou^2,#^, Jiayin Ling^1,3,#^, Yanbin Xu^,2,3,*^, Kailing Li^3^, Fei Wang^2^

#: Both authors contributed equally to this work.

*: Corresponding author: hopeybxu@163.com

1. Guangdong Provincial Key Laboratory of Environmental Health and Land Resource, Zhaoqing University, Zhaoqing, Guangdong, 526061, China

2. Analysis and Test Center, Guangdong University of Technology, Guangzhou, 510006, China

3. School of Environmental Science and Engineering, Guangdong University of Technology, Guangzhou, 510006, China

Table S1. Recoveries of the five analytes at three concentration levels (n=5)

| Compounds | Spiked concentration  (ng/mL) | Measure concentration  (ng/mL) | RE (%) | RSD (%) |
| --- | --- | --- | --- | --- |
| Lactic acid | 100 | 109.57 | 109.57 | 7.61 |
|  | 400 | 405.41 | 95.89 | 3.33 |
|  | 800 | 767.11 | 101.35 | 4.51 |
| Succinic acid | 50 | 58.19 | 116.39 | 6.12 |
|  | 100 | 112.42 | 112.42 | 7.34 |
|  | 200 | 206.25 | 103.12 | 4.66 |
| Acetophenone | 5 | 6.41 | 100.70 | 1.93 |
|  | 10 | 10.26 | 102.59 | 1.25 |
|  | 20 | 19.64 | 98.22 | 1.14 |
| Cinnamic acid | 50 | 52.26 | 104.53 | 4.69 |
|  | 100 | 105.07 | 105.07 | 9.80 |
|  | 200 | 206.26 | 103.47 | 9.32 |
| Phenyllactic acid | 5 | 5.11 | 102.36 | 0.57 |
|  | 10 | 11.06 | 110.58 | 0.77 |
|  | 20 | 22.73 | 113.64 | 1.12 |

RE: recovery

RSD: relative standard deviation

Fig. S1. The extract chromatogram and MS/MS of succinic acid in the sample (top) compared with reference standards (bottom)

Fig. S2. The extract chromatogram and MS/MS of acetophenone in the sample (top) compared with reference standards (bottom)

 Fig. S3. The extract chromatogram and MS/MS of cinnamic acid in the sample (top) compared with reference standards (bottom)

 Fig. S4. The extract chromatogram and MS/MS of phenyllactic acid in the sample (top) compared with reference standards (bottom)

Fig. S5. MS2 spectrum comparison between the sample and mzCloud library of Υ-aminobutyric acid (A), L-glutamic acid (B), proline (C) and D-(+)-pyroglutamic acid (D)

Fig. S6. The chromatogram and ms2 spectrum of extract mass 132.1019

Fig. S7. The chromatograph of lactic acid and succinic acid under 30°C (A) and 20°C (B)

**

Fig. S8. Calibration curves of the five analytes
